# Label-free quantitative 3D mapping of collagen architecture by holotomography

**DOI:** 10.64898/2026.05.05.722893

**Published:** 2026-05-08

**Authors:** Sehyeon Lee, Wei Sun Park, Juheon Lee, Juyeon Park, Hyeoncheol Park, Eun Hyun Ahn, Deok-Ho Kim, YongKeun Park

**Affiliations:** 1 Department of Physics, Korea Advanced Institute of Science and Technology (KAIST), Daejeon 34141, Republic of Korea;; 2 KAIST Institute for Health Science and Technology, KAIST, Daejeon 34141, Republic of Korea;; 3 Department of Biomedical Engineering, Johns Hopkins University, Baltimore, MD 21205, USA;; 4 Center for Microphysiological Systems, Johns Hopkins University, Baltimore, MD 21205, USA;; 5 Department of Medicine, Johns Hopkins University, Baltimore, MD 21205, USA;; 6 Tomocube Inc., Daejeon 34109, Republic of Korea.

## Abstract

Quantitative label-free analysis of collagen architecture lacks physically calibrated volumetric methods. Here we present holotomography (HT), which reconstructs three-dimensional refractive-index (RI) distributions of collagen networks. Because RI scales linearly with dry mass density, HT yields fiber-level morphology and mass from a single acquisition. HT resolves 3D fibrillar structure, distinguishes subtype-specific organizational and mass differences, and tracks polymerization and cell-driven remodeling, establishing a quantitative framework for extracellular matrix analysis.

Quantitative three-dimensional (3D) characterization of collagen architecture is central to understanding tissue mechanics, fibrosis and cancer invasion, yet no existing label-free imaging modality provides physically calibrated volumetric readouts of fibrillar collagen networks^1,2^. Fluorescence microscopy requires exogenous probes that can perturb matrix organization^3^. Second harmonic generation (SHG) microscopy detects fibrillar collagen without labels and probes molecular organization^4,5^ with polarization-resolved analysis, but its intensity depends on fiber orientation and provides limited cellular contrast. Confocal reflection microscopy (CRM) enables label-free fibrillogenesis monitoring^6^ but yields only qualitative scattering contrast. Two-dimensional quantitative phase imaging (QPI)^7^ and optical coherence tomography (OCT)^8^ provide phase or scattering contrast, yet neither delivers volumetric RI reconstruction with per-fiber physical readouts.

Here we present HT-based collagen characterization. HT, an optical diffraction tomography technique, reconstructs continuous 3D RI distributions from multiple illumination angles^9,10^. Because RI scales linearly with dry mass density^11^, the reconstructed maps yield morphological descriptors, orientation statistics and per-fiber dry mass from a single acquisition. Whereas prior QPI studies characterized collagen in two dimensions^7^, HT enables full volumetric reconstruction of intact 3D networks.

Reconstructed 3D RI tomograms (axial extent ~30 μm) revealed continuous fibrillar collagen architecture and resolved individual fibrils and their axial continuity without exogenous labeling (Fig. 1a). Co-registration with SHG microscopy confirmed spatial correspondence between HT-resolved fibrils and SHG-positive collagen (Fig. 1b). Further comparisons with SHG and FITC-collagen fluorescence are provided in Extended Data Figs. 2 and 3.

Volumetric RI renderings of separately prepared type I and type III collagen at matched and varied concentrations revealed distinct network organizations with increased fibrillar RI (Fig. 1c). To extract quantitative descriptors, we applied curvelet-based fiber segmentation (CT-FIRE^12^) to representative axial sections, generating binary fiber masks and computing effective width, fragment length, straightness and orientation angle (Fig. 1d). Effective-width-weighted masks enabled per-fiber dry mass estimation from local RI contrast^13^ (Fig. 1e). This quantification currently operates on 2D axial sections extracted from the 3D tomograms and extension to full 3D fiber tracing is straightforward.

RI probability density analysis, separated into fiber-associated and background regions, confirmed that fibrillar collagen occupies a distinct higher-RI population in both types (Fig. 1f). With increasing concentration, effective width and per-fiber dry mass shifted systematically upward, more strongly in type I than type III collagen (Fig. 1g), consistent with the thicker, more bundled fibril architecture characteristic of type I networks. Additional metric distributions appear in Extended Data Figs. 4 and 5. These data show that HT-derived metrics resolve morphological and dry-mass differences between type I and type III collagen networks at the single-fiber level. Discrimination of subtypes within mixed or tissue samples would require additional contrast such as polarization-resolved SHG^5^ or immunolabeling.

The label-free and non-phototoxic nature of HT also enables continuous time-resolved imaging. During type I collagen polymerization, mean RI change (Δ*n*) monotonically increased over time, consistent with progressive fibrillar assembly. This time-resolved RI trajectory provides a physically calibrated kinetic readout of matrix formation that complements earlier CRM- and SHG-based fibrillogenesis studies^6,14^ (Fig. 2a; Supplementary Video 1).

We performed time-lapse HT imaging of HT1080 fibrosarcoma cells^15^ embedded in collagen and treated with the ROCK inhibitor Y-27632 or broad-spectrum MMP inhibitor GM6001. Both treatments altered cell-associated matrix remodeling relative to vehicle controls, with spatially resolved changes captured at the single-fiber level (Fig. 2b, c). Matched controls and complementary datasets are provided in Extended Data Figs. 6–9 and Supplementary Videos 2–5. The continuous volumetric RI maps enables quantitative tracking of local matrix reorganization, complementing endpoint and intensity-based approaches.

These results establish HT as a label-free platform for quantitative collagen characterization spanning static architecture, assembly kinetics, and cell-driven remodeling. HT provides a physically calibrated RI map from which per-fiber dry mass and morphological descriptors are directly derived. This capability is not provided by SHG, which instead provides molecular-level organization of collagen^4,5^, or by CRM, which yields qualitative scattering contrast^6^. The two modalities are therefore complementary rather than competing. The underlying optical diffraction tomography principle can be implemented on any coherent imaging platform with multi-angle illumination, making the approach broadly applicable across hardware configurations.

Because RI contrast is not collagen-specific, HT measures total RI per voxel. In the reconstituted gels examined here, collagen dominates the high-RI signal. In complex tissues, isolating collagen from other high-RI constituents would require computational specificity or multimodal validation, and dense samples may introduce multiple-scattering artifacts^16^. Extension to 3D fiber tracing, tissue-derived matrices and additional cell systems will broaden HT-based ECM analysis toward fibrosis drug screening, engineered-tissue quality control and quantitative mechanobiology^17^.

## Methods

### Holotomography

**Extended Data Fig. 1 |:**
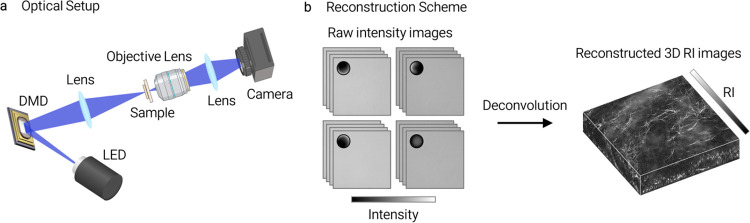
Holotomography imaging system and 3D RI reconstruction workflow. The HT platform acquires volumetric RI tomograms from multiple structured-illumination measurements. **a**, System schematic: low-coherence illumination modulated by a DMD is directed through the sample and recorded by a camera. **b**, Representative raw intensity images under sequential illumination conditions and reconstructed 3D RI tomogram showing spatially resolved fibrillar collagen structures.

Unless otherwise noted, refractive-index (RI) tomograms were acquired on a holotomography platform (HT-X1, Tomocube Inc.) using 450-nm LED illumination and digital-micromirror-device (DMD)-based structured illumination (Extended Data Fig. 1). The DMD modulates low-coherence illumination patterns^1–3^; multiple intensity images recorded under varying illumination conditions and axial positions are reconstructed into a 3D RI tomogram by an inverse-scattering algorithm.

For FITC-labeled type I collagen experiments, co-registered HT and fluorescence imaging were performed with the integrated confocal fluorescence module (HT-X1 Plus, Tomocube Inc.). Both modalities share the same optical path, so fluorescence images were acquired from the same field of view and aligned with the HT reconstructions in the *x*–*y* plane; axial range of the fluorescence z-stack was defined independently during acquisition.

### Second harmonic generation microscopy

Co-registered SHG imaging was performed on an IVIM-CMS two-photon microscope (IVIM Technology) with 920-nm excitation. The corresponding region of interest was manually identified after transferring the sample between the HT and SHG systems. Post-acquisition registration was performed in MATLAB using mutual-information-based rigid alignment on gradient-magnitude images (imregtform, imwarp), followed by affine refinement.

### Morphological descriptors

Following CT-FIRE-based fiber segmentation, fiber-level descriptors were computed for each extracted fiber. Fiber length is the cumulative path length along the traced centerline; fiber angle is the absolute orientation of the line joining the two endpoints. Straightness is the ratio of end-to-end distance to path length, with values closer to 1 indicating straighter fibers. Width was estimated from the extracted fiber geometry by CT-FIRE^4,5^.

### Dry mass calculation

Dry mass was estimated from RI maps using the linear relation between RI and non-aqueous biomolecule concentration established by Barer^6^ and applied in subsequent RI-tomography studies^7,8^. The local RI is expressed as *n* = *n*_m_ + *α C*, where *n*_m_ is the medium RI, *α* is the specific RI increment (mL/g) and *C* is the dry mass density. Local dry mass density is therefore obtained from the RI contrast Δ*n* = *n* − *n*_m_ as *C* = Δ*n* / *α*.

For each collagen condition, RI-derived dry mass was calculated on representative axial sections extracted from the 3D RI tomograms after CT-FIRE-based fiber extraction. Fragment-wise mean RI values were obtained from segmented fiber regions and the medium RI (*n*_m_ = 1.3370, consistent with PBS and cell culture medium) was subtracted to yield Δ*n*. The specific RI increment was set to *α* = 0.18 mL/g, appropriate for a protein-dominated system^9^. RI values below *n*_m_ were set to zero before conversion to suppress non-physical negative contributions arising from reconstruction noise.

Per-fragment dry mass was computed from the fragment-wise RI contrast and the mask-defined volume. The mask-defined volume was approximated as *N V*_vox_, where *N* is the number of pixels in the effective-width-weighted fragment mask and *V*_vox_ is the voxel-equivalent volume. Per-fragment dry mass was then obtained as *m = (C) V*_vox_ *N*. This approach follows the same physical principle used in previous QPI/ODT studies^7,8^, restricted here to CT-FIRE-derived fiber regions so that dry mass specifically reflects fiber-associated material.

### Sample preparation

#### Collagen matrices.

Type I collagen (rat tail, 4 mg/mL; Advanced BioMatrix, Cat. No. 5153) and type III collagen (human, 1 mg/mL; Advanced BioMatrix, Cat. No. 5021) were kept on ice before use. Collagen solutions were neutralized with 10× PBS (final 1×) and 0.1 M NaOH to pH 7.2–7.4, then cast onto the 20 × 20 mm imaging area of a TomoDish (Tomocube) in 60-μL layers. Type I and type III collagen were prepared at the indicated final concentrations for each experiment. Gels were polymerized at 37 °C for 1 h (type I) or 2 h (type III). After gelation, complete medium (DMEM supplemented with 10% FBS and 1% penicillin–streptomycin) was added, and all imaging was performed in this medium at 37 °C under 5% CO2. Under the HTX1 imaging conditions used here, the background refractive index of the complete medium was indistinguishable from that of PBS within measurement precision.

#### Cells and drug treatments.

HT1080 cells (ATCC, CCL-121) were prepared as a single-cell suspension and mixed with neutralized collagen at 8,000 cells per 60 μL (1.3 × 10^5^ cells/mL) before casting. For perturbation experiments, cells were pretreated for 1 h with Y-27632 dihydrochloride (15 μM; Tocris, Cat. No. 1254) or GM6001 (10 μM; GLPBio, Cat. No. GC14523–1MG), embedded in collagen, and imaged in the continued presence of the corresponding compound in complete medium. GM6001 was prepared in DMSO and used at a final concentration of 10 μM; control samples received the same final concentration of DMSO in complete medium without drug. Y-27632 was prepared in 1× PBS and used at a final concentration of 15 μM; control samples received the corresponding volume of PBS in complete medium without drug. Time-lapse datasets were acquired at 5-min intervals over 0–2 h and 24–26 h after gelation for the conditions shown in Extended Data Figs. 6–9.

#### FITC-collagen tracer.

For fluorescence-based comparison experiments, FITC-labeled type I collagen (Sigma-Aldrich, Cat. No. C4361) was mixed with unlabeled type I collagen at 5% (w/w) of the total collagen mass before neutralization and casting.

### Statistics and reproducibility

**Figure 1 |:**
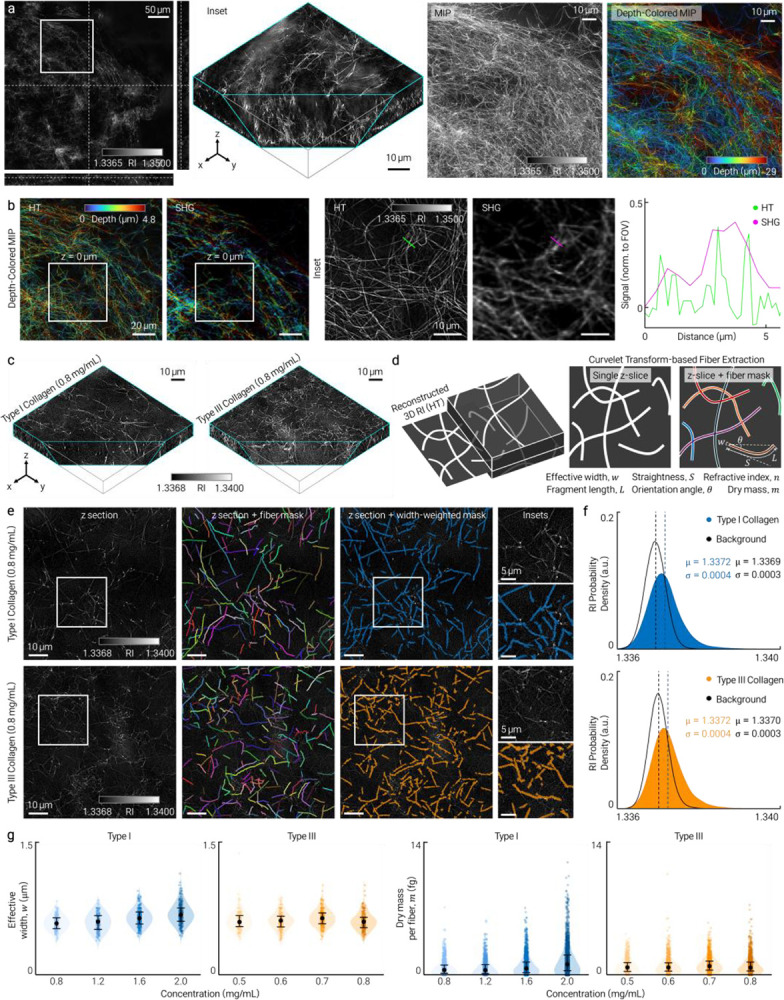
Label-free 3D RI imaging and quantitative characterization of collagen networks by holotomography. HT reconstructs volumetric RI distributions of fibrillar collagen without exogenous labels, enabling automated fiber-level quantification. **a**, Representative 3D RI tomogram (axial extent ~30 μm) visualized as orthogonal slices, volumetric rendering, maximum-intensity projection (MIP), and depth-colored MIP, resolving continuous fibrillar architecture. **b**, Co-registered HT and SHG images with corresponding line profiles, confirming spatial correspondence of individual fibrils. **c**, Volumetric RI renderings of separately prepared type I and type III collagen networks, highlighting distinct fibrillar organization. **d**, Quantitative analysis pipeline: HT construction followed by curvelet transform-based fiber segmentation (CT-FIRE) applied to representative axial sections, generating fiber masks and centerlines. **e**, Representative concentration-matched (0.8 mg/mL) sections of type I and type III collagen with extracted fibers and effective-width-weighted masks used for per-fiber quantification. **f**, RI probability density distributions for fiber-associated and background regions, showing distinct higher-RI populations corresponding to fibrillar collagen. **g**, Concentration-dependent distributions of effective fiber width and per-fiber dry mass for type I and type III collagen, demonstrating systematic shifts with increasing concentration and subtype-specific differences.

**Figure 2 |:**
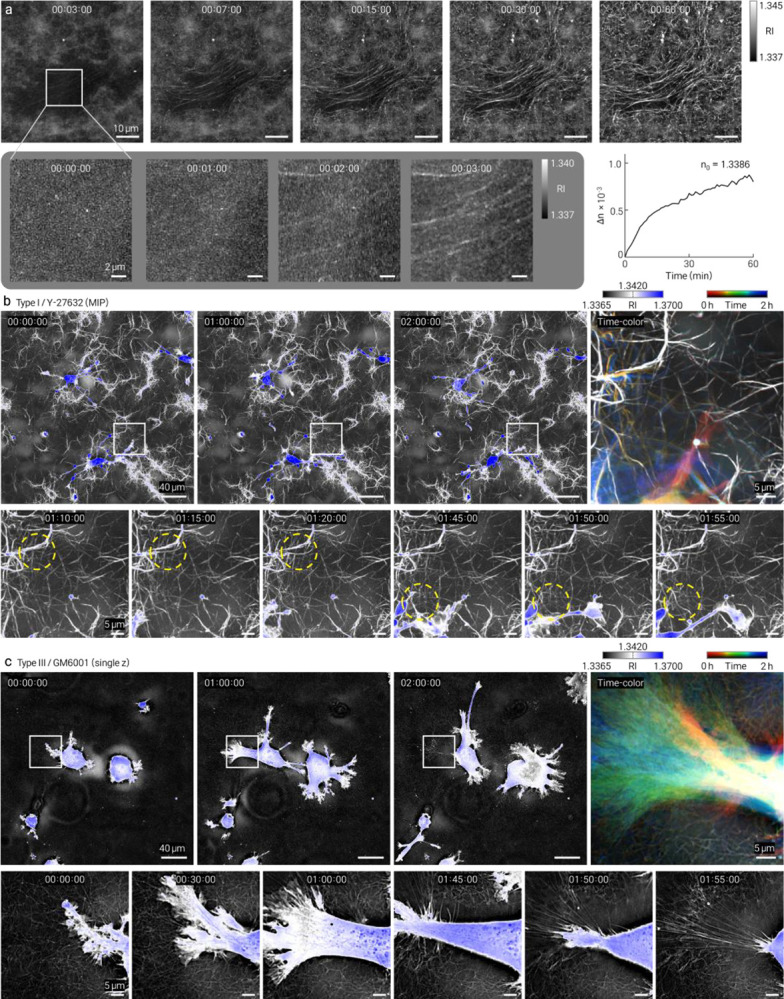
Time-resolved HT imaging of collagen assembly kinetics and drug-dependent cell–matrix remodeling. HT enables continuous, label-free monitoring of collagen dynamics through volumetric refractive-index (RI) mapping. **a**, Time-resolved HT MIPs of type I collagen polymerization showing progressive fibrillar assembly over time. Insets highlight early fibril emergence. Quantification of mean RI change (Δ*n*) demonstrates a monotonic increase during gelation, providing a physically calibrated readout of assembly kinetics. **b, c**, Time-lapse HT imaging of HT1080 cells embedded in collagen under pharmacological perturbation (5-min intervals). **b**, Type I collagen with ROCK inhibitor (Y-27632). **c**, Type III collagen with MMP inhibitor (GM6001). For each condition, panels show full-field views at representative time points (0, 1, and 2 h), temporally encoded composite images, and zoomed regions highlighting cell-associated matrix remodeling (dashed circles). Drug treatments alter local collagen organization and remodeling dynamics at the single-fiber level compared to controls.

For static collagen characterization (Fig. 1), representative axial sections were selected from independently prepared gels for each subtype and concentration condition. Fiber-level descriptors and RI-derived dry-mass were computed for all fibers extracted by CT-FIRE within each field of view; distributions in Fig. 1f, g represent pooled single-fiber measurements, with fiber counts reported in the source data. For time-lapse experiments (Fig. 2), representative datasets from independent gel preparations are shown for each drug and vehicle condition. No statistical method was used to predetermine sample size. Experiments were not randomized and investigators were not blinded to allocation during experiments or outcome assessment.

## Extended Data

**Extended Data Fig. 2 |:**
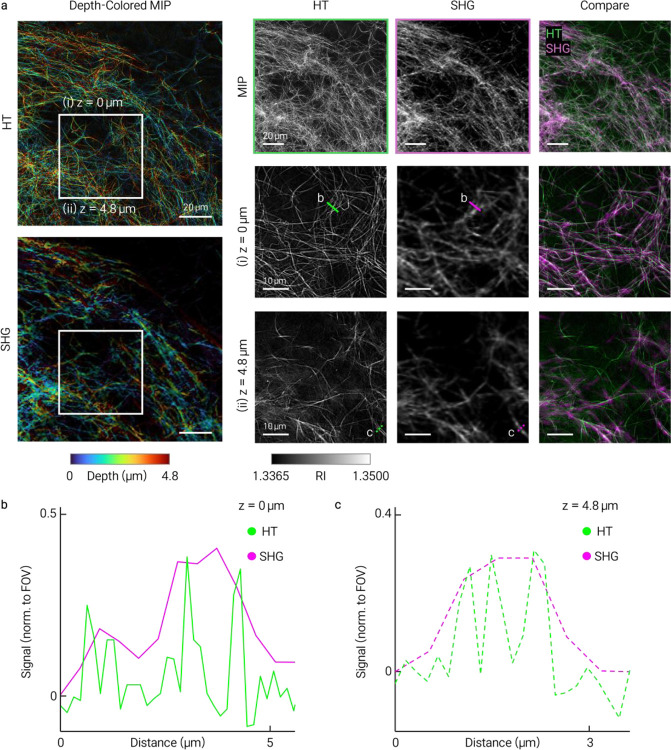
Axial correspondence between HT and SHG imaging of collagen networks. Co-registered HT and SHG images across multiple axial planes confirm structural correspondence. **a**, Depth-colored MIPs, conventional MIPs, grayscale views and merged overlays (HT, green; SHG, magenta). Inset regions at **(i)** *z* = 0 μm and **(ii)** *z* = 4.8 μm are enlarged. **b,c**, HT and SHG line profiles along marked segments in **a(i)** and **a(ii)**; solid and dashed lines denote the two axial planes. Broader SHG peaks are resolved as more separated peaks in HT, consistent with improved local structural delineation.

**Extended Data Fig. 3 |:**
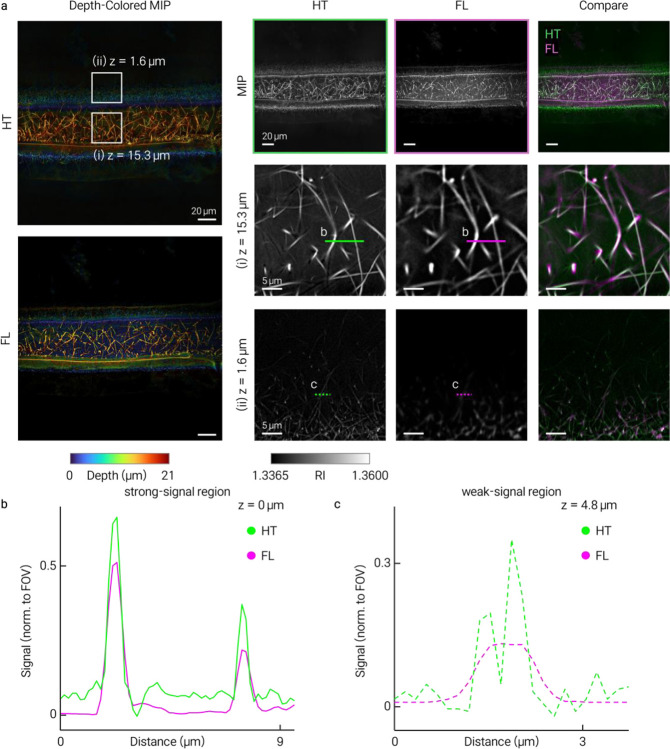
Co-registration of HT and fluorescence imaging of FITC-labeled collagen. HT and fluorescence (FL) images of FITC-labeled type I collagen compared across axial planes. **a**, Depth-colored views, grayscale images and merged overlays (HT, magenta; FL, green). Insets at **(i)** *z* = 15.3 μm and **(ii)** *z* = 1.6 μm. **b,c**, Line profiles along marked segments. In the strong-signal region (i), HT and FL show closely matched peaks; in the weak-signal region (ii), fibrillar features remained more clearly resolved in HT whereas FL exhibited lower local signal contrast.

**Extended Data Fig. 4 |:**
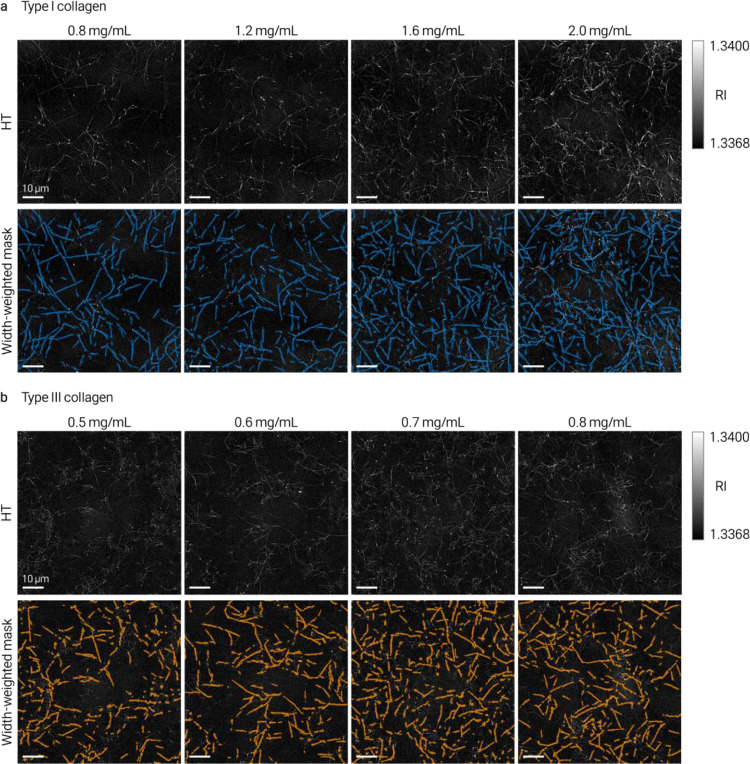
Concentration-series fibril extraction and mask generation in type I and type III collagen. Representative axial sections and effective-width-weighted masks across the full concentration series. **a**, Type I collagen. **b**, Type III collagen. The pipeline generated masks that followed the underlying fibrillar RI signal across all tested concentrations.

**Extended Data Fig. 5 |:**
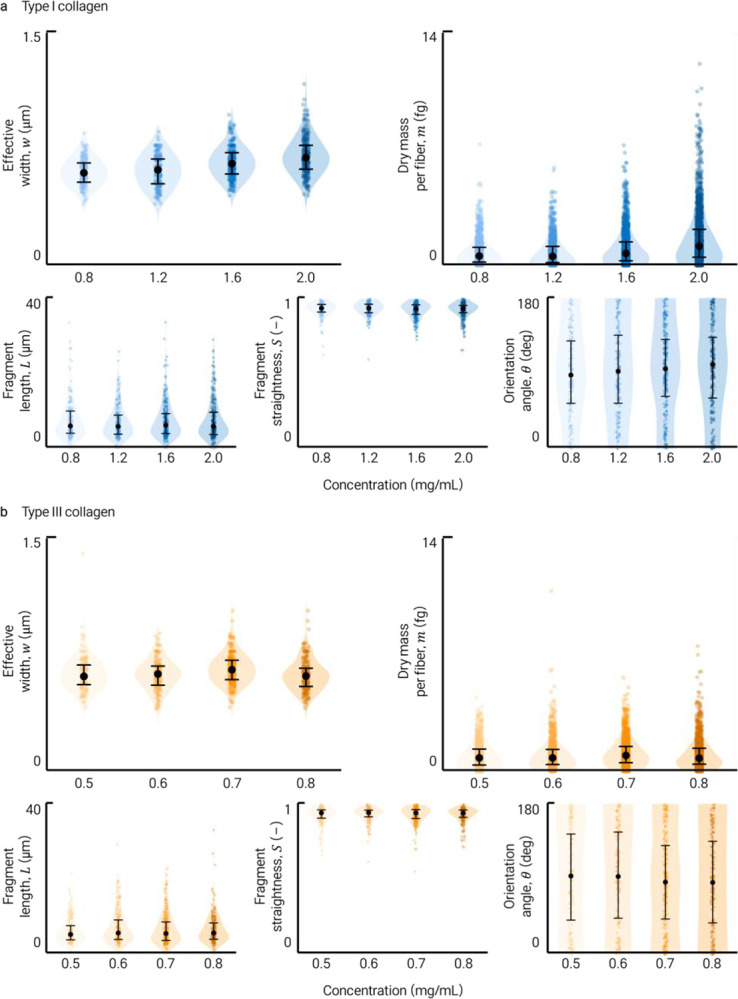
Full distributions of HT-derived fibrillar metrics across concentrations. Distributions of effective width, per-fiber dry mass, fragment length, straightness and orientation angle across the concentration range. **a**, Type I collagen. **b**, Type III collagen. In type I collagen, effective width and per-fiber dry mass shifted upward with increasing concentration, with increasingly right-skewed dry mass distributions. These changes were less pronounced in type III collagen, consistent with its finer network organization. Orientation angle remained broadly distributed in all conditions.

**Extended Data Fig. 6 |:**
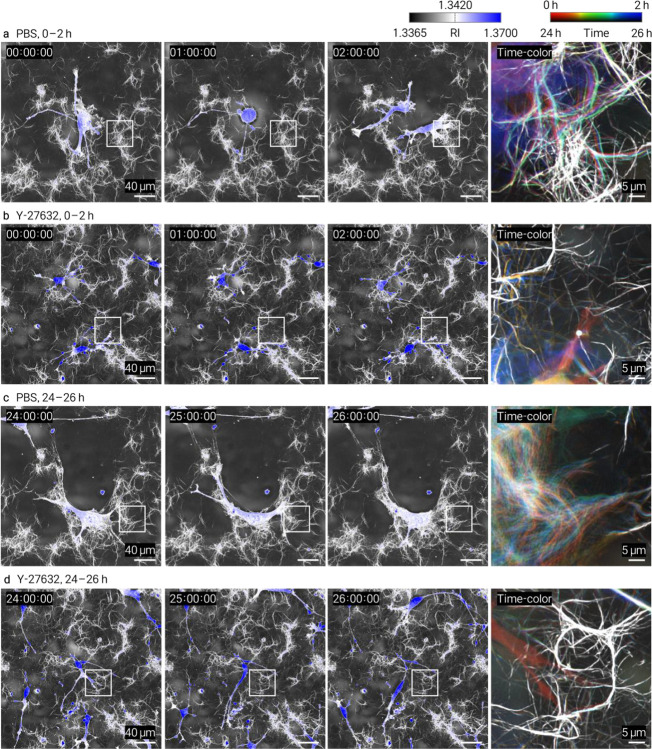
Time-resolved HT imaging of HT1080 cells in type I collagen under PBS or Y-27632 treatment. Each condition is shown at early (0–2 h) and late (24–26 h) time windows. **a**, PBS control, 0–2 h. **b**, Y-27632, 0–2 h. **c**, PBS control, 24–26 h. **d**, Y-27632, 24–26 h. Images are maximum-intensity projections; the RI colormap combines grayscale (collagen) with white-to-blue (cell-associated regions). Time-color images are temporally encoded RGB composites.

**Extended Data Fig. 7 |:**
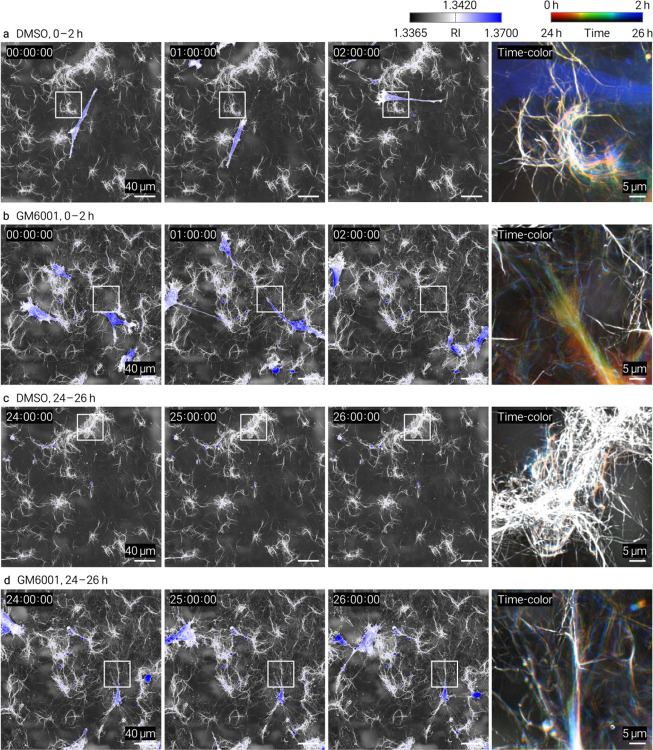
Time-resolved HT imaging of HT1080 cells in type I collagen under DMSO or GM6001 treatment. Shown in the same layout as in Extended Data Fig. 6. **a**, DMSO control, 0–2 h. **b**, GM6001, 0–2 h. **c**, DMSO control, 24–26 h. **d**, GM6001, 24–26 h. In **c**, no cell body was present in the field of view; the composite reflects off-frame cellular activity originating outside the imaged region.

**Extended Data Fig. 8 |:**
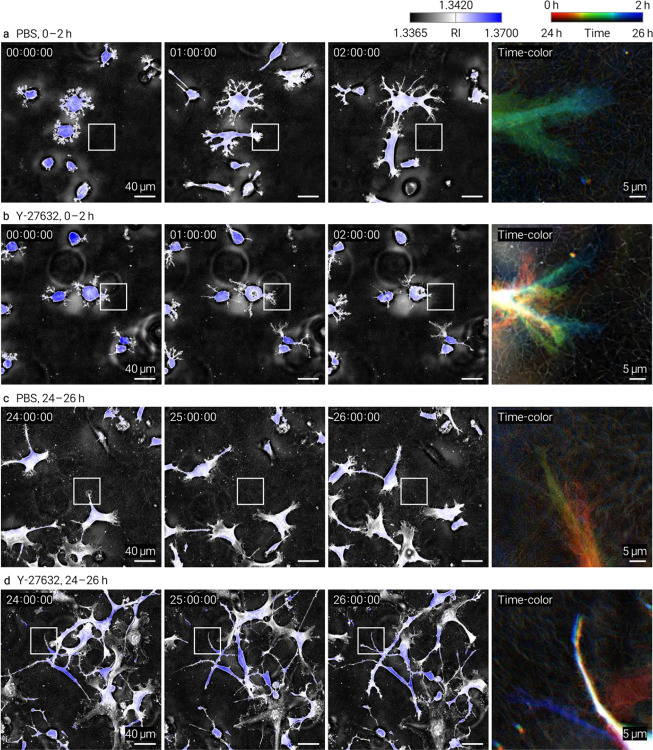
Time-resolved HT imaging of HT1080 cells in type III collagen under PBS or Y-27632 treatment. Each condition is shown at early (0–2 h) and late (24–26 h) time windows. **a**, PBS control, 0–2 h. **b**, Y-27632, 0–2 h. **c**, PBS control, 24–26 h. **d**, Y-27632, 24–26 h. For each panel, full-field single-z sections are displayed at representative time points alongside a temporally encoded composite of the boxed region. Single-z sections are shown to better resolve fibrillar features in the finer type III collagen network.

**Extended Data Fig. 9 |:**
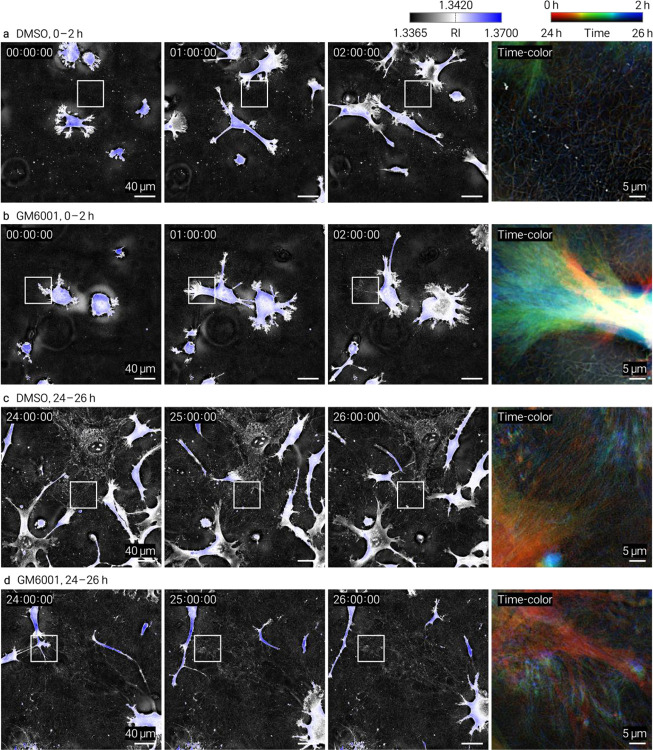
Time-resolved HT imaging of HT1080 cells in type III collagen under DMSO or GM6001 treatment. Shown in the same layout as in Extended Data Fig. 8. **a**, DMSO control, 0–2 h. **b**, GM6001, 0–2 h. **c**, DMSO control, 24–26 h. **d**, GM6001, 24–26 h.

## Data Availability

A representative subset of the imaging data is available on figshare. The full datasets are available upon request. Source data are provided with this paper.

## Code availability

Code used to generate the findings of this study is available at https://github.com/BMOLKAIST
